# Prevalence, characteristics, and costs of diagnosed homocystinuria, elevated homocysteine, and phenylketonuria in the United States: a retrospective claims-based comparison

**DOI:** 10.1186/s12913-020-5054-5

**Published:** 2020-03-06

**Authors:** Marcia Sellos-Moura, Frank Glavin, David Lapidus, Kristin Evans, Carolyn R. Lew, Debra E. Irwin

**Affiliations:** 1 Orphan Technologies, 430 Bedford St, Lexington, MA 02420 USA; 2 LapidusData Inc, 321 NE 4th St, Oklahoma City, OK 73104 USA; 3 IBM Watson Health, 75 Binney St, Cambridge, MA 02142 USA

**Keywords:** Homocysteine, Homocystinuria, Phenylketonuria, Prevalence, Costs, Utilization, Comorbidities, Medications

## Abstract

**Background:**

Classical homocystinuria (HCU), an inborn error of homocysteine metabolism, has previously been estimated to affect approximately 1 in 100,000–200,000 people in the United States (US). HCU is poorly detected by newborn screening, resulting in underestimates of its prevalence. This study compared characteristics, healthcare use and costs, and projected prevalence between patients with diagnosed HCU, elevated total homocysteine (tHcy), and diagnosed phenylketonuria (PKU).

**Methods:**

Patients in the MarketScan® Research Databases were identified with strictly-defined HCU (> 2 diagnoses, including 1 ICD-10), broadly-defined HCU (> 1 ICD-10), elevated tHcy (> 20 μmol/L) without an HCU diagnosis, or > 1 ICD-9/ICD-10 PKU diagnosis during 1/1/2010–12/31/2016 (first qualifying claim = index). Demographics and healthcare utilization and costs per patient per month (PPPM) were compared between all cohorts, frequencies of comorbidities and medications were compared between HCU and elevated tHcy patients, and healthcare provider types were assessed among HCU patients. The prevalence of patients meeting each cohort definition was projected to the United States (US) population.

**Results:**

Patients with strictly-defined (*N* = 2450) and broadly-defined (*N* = 6613) HCU, and with elevated tHcy (*N* = 2017), were significantly older than PKU patients (*N* = 5120) (57 vs. 56 vs. 53 vs. 18 years; *p* < 0.05). Vitamin D deficiency, hyperlipidemia, folic acid/B vitamins, and lipid-lowering medications, among others, were more common among diagnosed HCU patients vs. those with elevated tHcy (all *p* < 0.05). Rates of healthcare utilization were generally higher among HCU and elevated tHcy patients, compared to PKU, though total healthcare costs were similar between groups. Most HCU patients (~ 38%) received their index diagnosis from a primary care physician; very few (~ 1%) had any claim from a geneticist during their enrollment. The age-adjusted national prevalence of HCU was projected at 31,162 (95% CI: 30,411 – 31,913; ~ 1 in 10,000 of the US population) using the broad definition.

**Conclusions:**

The actual prevalence of HCU may be > 10 times prior estimates, at 1 in 10,000 in the US, and this study suggests that HCU is not being diagnosed until later in life. Improvements to newborn screening, detection in young children, and physician education regarding HCU among patients may be necessary to alleviate the burden of this genetic disease.

## Background

Classical homocystinuria (HCU), caused by mutations in the cystathionine beta synthase (CBS) gene, is an inherited genetic disorder in which the body is unable to metabolize the amino acid homocysteine (Hcy), a key molecule in several metabolic processes [1]. Deficiency of the CBS enzyme leads to elevated tissue and plasma levels of Hcy and its precursor, methionine [2]. In turn, patients can manifest symptoms ranging in severity from mild to severe involving the ocular, skeletal, vascular, and central nervous systems [3].

Published literature previously estimated the prevalence of HCU to be approximately 1 in 200,000–335,000 worldwide, and 1 in 100,000–200,000 in the United States (US) [4–6]. These estimates were largely based on patients seen by metabolic geneticists and diagnosed by newborn screening. Reports of the expected population frequency of likely pathogenic variants of CBS in European populations have suggested that this prevalence is underestimated [7,8]. The preferred newborn screening technique for HCU used in the US has poor sensitivity, as it tests for the precursor amino acid methionine rather than homocysteine. As methionine is an amino acid that can only be sourced from the diet, newborns at the time of screening may not have been exposed to sufficient methionine to detect abnormal levels. In addition, the cut-off values for elevated methionine are believed not to be sufficiently stringent. Taken together, it is widely acknowledged that newborn screening for HCU in the US likely leads to a significant number of false negatives [9]. Many patients are not diagnosed until they present with clinical events later in life (ocular disorders, developmental delays, or cardiovascular events including strokes, pulmonary embolism, and myocardial infarction at a young age), and some are likely never diagnosed [6].

The purpose of this study was to use a nationally representative administrative claims dataset to develop a better understanding of the population of patients with a confirmed diagnosis of HCU and those with severely elevated total Hcy (tHcy) without a record of HCU diagnosis, who may represent an undiagnosed segment of the HCU population. We described the demographic and clinical characteristics of patients with diagnosed HCU (strictly- and broadly-defined), patients with elevated tHcy but no HCU diagnosis, and a comparator group of patients diagnosed with phenylketonuria (PKU), another inborn error of amino acid (phenylalanine) metabolism where the majority of patients are detected by newborn screening at birth using a direct assay for phenylalanine. Additionally, we evaluated healthcare resource use and costs of patients diagnosed with HCU, patients with elevated tHcy, and patients diagnosed with PKU. Finally, we estimated the national prevalence of each condition (diagnosed HCU, elevated tHcy without an HCU diagnosis, and diagnosed PKU), and determined that the actual prevalence of HCU may be 1 in 10,000, which is 10 or more times current estimates of HCU prevalence, in agreement with prevalence estimates expected from the frequency of likely pathogenic CBS variants.

## Methods

### Data sources

Three IBM® MarketScan® Research Databases were used for this retrospective administrative claims study: the Commercial Database, the Medicare Supplemental Database, and the Lab Database. The Commercial Database contains health insurance claims across the continuum of care (e.g., inpatient, outpatient, outpatient pharmacy) as well as enrollment data from large employers and health plans across the US who provide private healthcare coverage for employees, their spouses, and dependents. This administrative claims database includes a variety of fee-for-service, preferred provider organizations (PPO), and capitated health plans. The Medicare Supplemental Database contains the same data as the Commercial Database for individuals with Medicare supplemental insurance paid for by employers. Both the Medicare-covered portion of payment (represented as Coordination of Benefits Amount) and the employer-paid portion are included in this database. Combined, the Commercial and Medicare Supplemental Databases contain over 100 million covered lives between 2010 and 2016 (the years included in the current study). The Lab Database contains laboratory results for a subset of patients contained in the MarketScan® Commercial and Medicare Supplemental Databases, including approximately 8 million covered lives between 2010 and 2016.

The MarketScan Research Databases were accessed via IBM MarketScan Treatment Pathways®, an online analytic interface that allows users to query the databases to identify patients with specific diagnoses, test results, procedures, and other clinical events, as well as to quantify healthcare resource use and costs. All study cohorts and variables were created and analyzed using the Treatment Pathways tool.

### Study population

Prior to the implementation of the International Classification of Diseases, Tenth Revision (ICD-10) coding system on October 1, 2015, the only diagnosis code designated to HCU in the ICD-Ninth Revision (ICD-9) was non-specific and was also used to indicate diagnoses of other conditions related to inborn errors of sulfur-bearing amino acid metabolism (ICD-9 code 270.4). After implementation of the ICD-10 coding system, a code specific to HCU (E722.11) and distinct from codes for MTHFR and cobalamin disorders became available. Therefore, we applied the following criteria to identify cohorts of strictly- and broadly-defined HCU.

Patients meeting the strictly-defined HCU criteria included those with > 2 non-diagnostic claims, > 90 days apart, with ICD-10 code E72.11, or patients with both > 1 non-diagnostic claim with ICD-9 code 270.4 and > 1 non-diagnostic claim with ICD-10 code E72.11 between January 1, 2010, and December 31, 2016. Patients meeting the broadly-defined HCU criteria included those meeting the strictly-defined HCU criteria as well as any patients with at least 1 non-diagnostic claim with ICD-10 code E72.11 between January 1, 2010 and December 31, 2016. The date of the first qualifying ICD-9 or ICD-10 diagnosis was the index date. Patients included in the strictly-defined HCU cohort are also a subset of the patients contained within the broadly-defined cohort.

Two additional cohorts were identified: the elevated tHcy cohort and the PKU cohort. The elevated tHcy cohort included patients who had > 1 tHcy lab result of > 20 μmol/L (the 98th percentile of the normal expected frequency distribution for tHcy) between January 1, 2010 and December 31, 2016 but were not in either the strictly- or broadly-defined HCU cohorts. The date of the first qualifying lab result was the index date. The PKU cohort included patients with > 1 non-diagnostic claim with ICD-9 diagnosis code 270.1 (phenylketonuria) or ICD-10 code E70.0 (classical phenylketonuria) between January 1, 2010 and December 31, 2016. The date of the first qualifying ICD-9 or ICD-10 PKU diagnosis during the study period was the index date. Since this is a prevalence sample, the first diagnosis during the study period does not necessarily represent the first time the patient was diagnosed with either HCU or PKU, nor does it represent the first time the patient may have had an elevated tHcy lab value.

### Patient characteristics

All demographic characteristics available in the MarketScan Databases were measured on the index date and included age, sex, geographic region, urban residence, and insurance plan type (race/ethnicity is not available in the databases). The clinical characteristics (comorbid diagnoses and medications) measured were chosen because they were known to be potentially associated with HCU or with other conditions leading to elevated tHcy levels. These characteristics were measured in both the strictly-defined and the broadly-defined HCU diagnosis groups and also in those with tHcy levels suggestive of HCU but who did not have an HCU diagnosis. These characteristics were not measured in the PKU group since they are not necessarily considered to be associated with PKU. Since HCU is an inborn error of metabolism, the presence of comorbid conditions and medication use was assessed based on claims during all of a patient’s enrollment period in the database between January 1, 2010, and July 31, 2017. The presence of comorbidities was defined as > 1 non-diagnostic medical claim with an ICD-9 or ICD-10 diagnosis code for the following conditions: anxiety/depression, chronic kidney disease/kidney failure, coagulation disorders, developmental/intellectual disabilities, type 2 diabetes, essential hypertension, foot/knee/chest deformity, hemiplegia/paraplegia, hyperlipidemia, hypothyroidism, joint/limb pain, lens dislocation, Marfan syndrome, megaloblastic anemia, myocardial infarction, neurological manifestations, osteoporosis, retinal detachment, scoliosis, stroke/TIA, non-stroke thrombosis, vitamin D deficiency. Medication use was defined as > 1 outpatient pharmacy claim for any drug in the following therapeutic classes: blood thinners/thrombolytics, antidiabetics, antihypertensives, anxiolytics/antidepressants, folic acid or other B vitamins, lipid-lowering drugs, other cardiovascular drugs, pain medications, steroids, thyroid hormones, vitamin D.

### Healthcare provider type

The type of healthcare provider on the index HCU diagnosis claim was also identified for strictly- and broadly-defined HCU patients. In addition, we examined whether the healthcare provider noted on any claim during HCU patients’ enrollment in MarketScan between January 1, 2010 and July 31, 2017 was a genetics specialist, and tabulated the proportion of patients who had ever seen a genetics specialist in order to estimate the proportion of the patients that may have been referred to a geneticist for inborn genetic conditions.

### Healthcare resource utilization and costs outcomes

Healthcare resource use and costs were assessed for patients in each cohort from the index date through either the end of the patient’s MarketScan enrollment or December 31, 2016, whichever came first. Since each patient has a different amount of continuous enrollment after their index date, a variable-length follow-up period was assessed for healthcare resource use and costs. Data were collected on the percentage of patients with ≥1 claim, number of claims or visits per patient per month (PPPM), and costs PPPM for the following service categories: inpatient (IP) admissions, emergency room (ER) visits, outpatient (OP) office visits, laboratory tests, other OP visits (radiology and other medical services outside of a physician’s office that are not captured in the other categories), and OP pharmacy. Total medical costs PPPM, including all IP and OP medical services, were also calculated, as were total healthcare costs (medical + OP pharmacy costs). Utilization and costs were calculated PPPM due to the variability in follow-up time between patients. Unadjusted healthcare costs include the paid amounts of fully adjudicated claims, including insurer and health plan payments as well as patient out-of-pocket costs.

### National Prevalence Estimates

The projected prevalence of strictly- and broadly defined diagnosed HCU, elevated tHcy without an HCU diagnosis, and diagnosed PKU were estimated by first stratifying the study population of HCU patients found in the MarketScan database and the overall population in the MarketScan database by the following age groups: 0–11, 12–17, 18–24, 25–34, 35–44, 45–54, 55–64, 65–74, 75–84, 85+. Prevalence rates in MarketScan per 1000 were then calculated by age group. The US population on July 1, 2016 (*N* = 323,127,513), stratified by the same age group categories, was obtained from the US Census Bureau [10]. The MarketScan prevalence rate for each age group was then applied to the US population age strata to estimate the national prevalence by age category. The prevalence estimates for each age group were then summed to estimate the age-adjusted national prevalence of diagnosed HCU, elevated tHcy without an HCU diagnosis, and diagnosed PKU.

### Data analysis

T-tests were used to compare the means of continuous variables (age, number of healthcare encounters, healthcare costs) between patients with diagnosed HCU, elevated tHcy, and diagnosed PKU. Chi-squared tests were used to compare the proportions of patients in each category of the other demographic characteristics, patients with each diagnosis and medication of interest, and patients with > 1 of each type of healthcare encounter.

## Results

### Patient selection

**Fig. 1:**
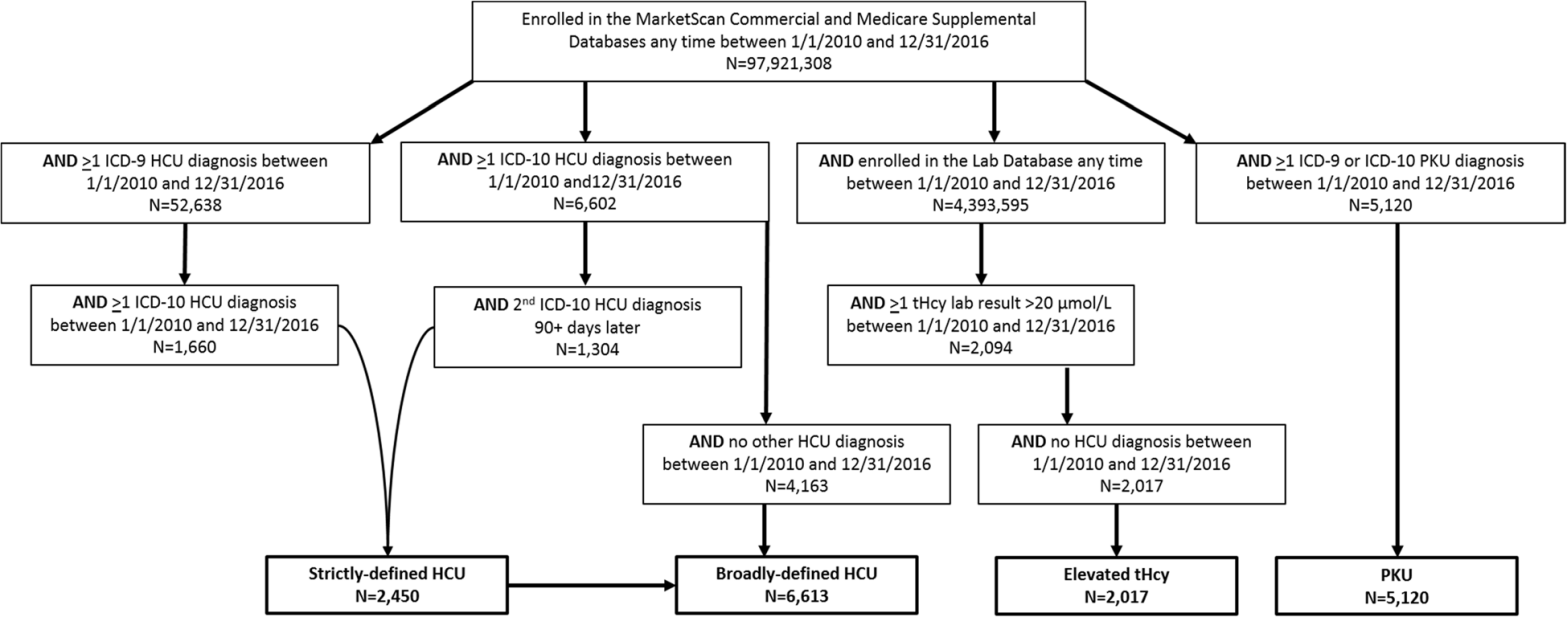
Selection of patients with diagnosed HCU, elevated tHcy without diagnosed HCU, and diagnosed PKU. ICD: International Classification of Diseases; HCU: homocystinuria; PKU: phenylketonuria; tHcy: total homocysteine

Out of 100.5 million patients in the Commercial and Medicare databases during the study period, a total of 2450 patients met the criteria for the strictly-defined HCU cohort (> 2 HCU diagnoses, > 1 of these diagnosis being an ICD-10 diagnosis) and an additional 4163 patients had only 1 claim with an HCU diagnosis and that claim had to be an ICD-10 diagnosis. This resulted in 6613 total patients in the broadly-defined HCU cohort. Out of 8 million patients with data in the Lab Database during the study period, 2094 patients had > 1 tHcy lab result > 20 μmol/L between January 1, 2010 and December 31, 2016. Over 96% of the patients in the Lab Database with an elevated tHcy test result had no claims with an ICD-9 or ICD-10 HCU diagnosis during that time period (*n* = 2017), and these are the patients that comprised the cohort of patients with elevated tHcy without an HCU diagnosis. Between January 1, 2010 and December 31, 2016, 5120 patients had > 1 non-diagnostic claim with either an ICD-9 or ICD-10 diagnosis code for PKU and were included in the PKU cohort (Fig. 1).

### Demographic characteristics

Not surprisingly given the effectiveness of newborn screening for PKU and the limitations of newborn screening for HCU, patients with an HCU diagnosis and those with elevated tHcy were significantly older at the time of their index claim, compared to patients with diagnosed PKU (strictly-defined HCU: mean [SD] = 56.8 [14.6]; broadly-defined HCU: 55.5 [14.8]; elevated tHcy: 52.8 [15.0]; PKU: 17.5 [21.0] years; *p* < 0.05; Table 1). More than half of PKU patients were younger than 12 years old at the time of their first recorded PKU claim, while most strictly- and broadly-defined HCU and elevated tHcy patients (74–84%) were 45 or older at the time of their first HCU diagnosis or elevated tHcy test result in the database between January 1, 2010 and December 31, 2016. Patients with a strictly- or broadly-defined HCU diagnosis were slightly but significantly more likely to be male (strictly-defined HCU: 52%; broadly-defined HCU: 49%), compared to patients with elevated tHcy (44%; *p* < 0.05) and compared to patients with PKU (47%; *p* < 0.05), and patients with a strictly- or broadly-defined HCU diagnosis or elevated tHcy were significantly more likely to live in an urban area (strictly-defined HCU: 91%; broadly-defined HCU: 90%; elevated tHcy: 93%), compared to PKU patients (85%; *p* < 0.05). Most patients in each cohort were covered by an EPO or PPO health plan, though this plan type was significantly more common among PKU patients (64%), compared to strictly-defined (57%) and broadly-defined (56%) HCU patients and patients with elevated tHcy (54%; all p < 0.05).

**Table 1:** Demographic characteristics of patients with strictly- and broadly-defined HCU, elevated tHcy without HCU, and PKU

	Strictly-defined HCU^**a**^	Broadly-defined HCU^**b**^	Elevated tHcy^**c**^	PKU^**d**^
	*N* = 2450	*N* = 6613	*N* = 2017	*N* = 5120
**Age (mean, SD)**	56.8 (14.6)*^	55.5 (14.8)*^	52.8 (15.0)*	17.5 (21.0)
**Age groups**				
0–11	18 (0.7%)*	34 (0.5%)*	11 (0.5%)*	2734 (53.4%)
12–17	19 (0.8%)*	49 (0.7%)*	21 (1.0%)*	439 (8.6%)
18–24	33 (1.3%)*^	119 (1.8%)*	45 (2.2%)*	381 (7.4%)
25–34	82 (3.3%)*^	339 (5.1%)*^	153 (7.6%)*	569 (11.1%)
35–44	245 (10.0%)*^	778 (11.8%)*^	291 (14.4%)*	348 (6.8%)
45–54	590 (24.1%)*	1596 (24.1%)*	528 (26.2%)*	232 (4.5%)
55–64	845 (34.5%)*	2227 (33.7%)*	650 (32.2%)*	211 (4.1%)
65–74	349 (14.2%)*^	810 (12.2%)*^	155 (7.7%)*	121 (2.4%)
75–84	193 (7.9%)*^	480 (7.3%)*^	115 (5.7%)*	58 (1.1%)
85+	76 (3.1%)*	181 (2.7%)*	48 (2.4%)*	27 (0.5%)
**Male**	1266 (51.7%)*^	3254 (49.2%)*^	893 (44.3%)*	2406 (47.0%)
**Region**				
Northeast	299 (12.2%)*^	820 (12.4%)*^	409 (20.3%)*	906 (17.7%)
North Central	681 (27.8%)*^	1574 (23.8%)*^	347 (17.2%)*	1014 (19.8%)
South	992 (40.5%)*^	2877 (43.5%)*^	1075 (53.3%)*	1751 (34.2%)
West	474 (19.3%)*^	1323 (20.0%)*^	185 (9.2%)*	1388 (27.1%)
Missing	4 (0.1%)*	20 (0.3%)*^	1 (0.04%)*	61 (1.2%)
**Urban residence**	2219 (90.6%)*^	5978 (90.4%)*^	1880 (93.2%)*	4357 (85.1%)
**Insurance type**				
Comprehensive	353 (14.4%)*^	853 (12.9%)*^	175 (8.9%)*	138 (2.7%)
HMO	155 (6.3%)*^	430 (6.5%)*^	249 (12.3%)	558 (10.9%)
EPO/PPO	1402 (57.2%)*^	3710 (56.1%)*	1088 (53.9%)*	3277 (64.0%)
POS/POS w/ capitation	190 (7.8%)*	489 (7.4%)	152 (7.5%)	333 (6.5%)
Other	301 (12.3%)*^	979 (14.8%)*	301 (14.9%)*	507 (9.9%)
Unknown	49 (2.0%)*	145 (2.2%)*	52 (2.6%)*	307 (6.0%)

^a^ > 2 HCU diagnoses, including > 1 ICD-10 diagnosis; ^b^ > 1 ICD-10 HCU diagnosis; ^c^ tHcy lab result > 20 μmol/L; ^d^ > 1 ICD-9 or ICD-10 PKU diagnosis; EPO/PPO: exclusive provider organization/preferred provider organization; *HCU* homocystinuria, *HMO* health maintenance organization, *PKU* phenylketonuria, *POS* point of service, *SD* standard deviation, *tHcy* total homocysteine; *significantly different from PKU, *p* < 0.05; ^significantly different from elevated tHcy, *p* < 0.05

### Clinical characteristics

**Fig. 2:**
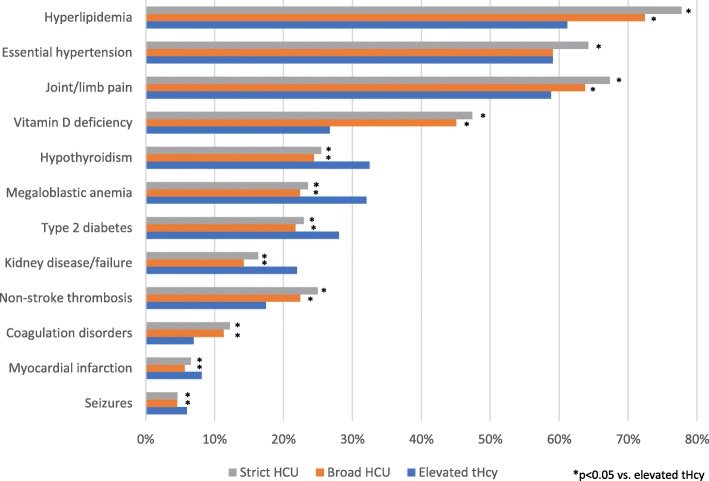
Prevalence of select comorbidities among patients with diagnosed HCU and elevated tHcy without diagnosed HCU. HCU: homocystinuria; tHcy: total homocysteine

The clinical characteristics measured were chosen because they were known to be potentially associated with HCU or with other conditions leading to elevated tHcy levels and were measured in both the HCU diagnosis groups and in the cohort with tHcy levels suggestive of HCU. In general, the prevalence of comorbid conditions was similar between the strictly- and broadly defined HCU cohorts. However, several comorbidities were significantly more prevalent among patients with an HCU diagnosis (either strictly- or broadly- defined), compared to those without an HCU diagnosis but with elevated tHcy, including vitamin D deficiency (strictly-defined HCU: 47% and broadly-defined HCU: 45% vs elevated tHcy: 27%), coagulation disorders (12 and 11% vs. 7%), non-stroke thrombosis (25 and 22% vs. 18%), hyperlipidemia (78 and 72% vs. 61%), and joint/limb pain (67 and 64% vs. 59%) (all *p* < 0.05; Fig. 2).

Some diagnoses associated with other conditions leading to elevated tHcy levels were more common among patients with elevated tHcy compared to patients with strictly- or broadly defined HCU, including kidney disease/failure (elevated tHcy: 22% vs. strict HCU: 16% and broad HCU: 14%), megaloblastic anemia (32% vs. 24 and 22%), hypothyroidism (33% vs. 26 and 24%), myocardial infarction (8% vs. 7 and 6%), and type 2 diabetes (28% vs. 23 and 22%%) (all *p* < 0.05; Fig. 2).

**Fig. 3:**
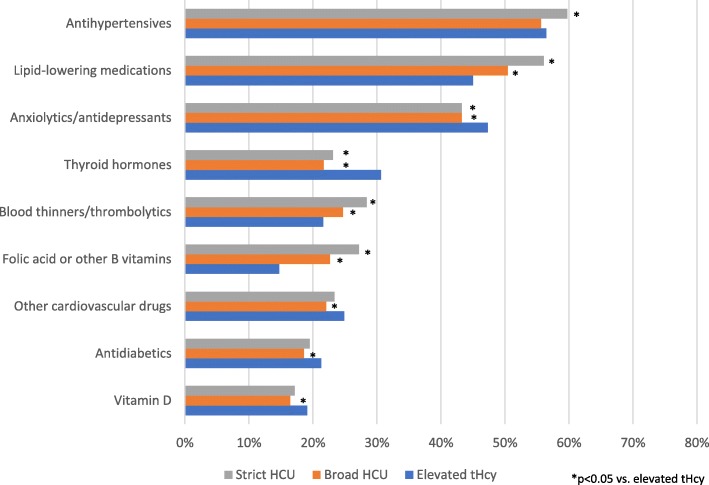
Prevalence of medication classes among patients with diagnosed HCU and elevated tHcy without diagnosed HCU. HCU: homocystinuria; tHcy: total homocysteine

The prevalence of some prescriptions was significantly higher among patients with either a strictly- or broadly-defined HCU diagnosis, compared to those with elevated tHcy, including folic acid and other B vitamins (strictly-defined HCU: 27% and broadly-defined HCU: 23% vs. elevated tHcy: 15%), blood thinners/thrombolytics (28 and 25% vs. 22%), and lipid-lowering medications (56 and 51% vs. 45%) (all *p* < 0.05; Fig. 3) Thyroid hormone prescriptions were significantly more prevalent among patients with elevated tHcy, compared to patients with a strictly- or broadly-defined HCU diagnosis (31% vs. 23 and 22%), as were prescriptions for vitamin D (19% vs. 17 and 16%), certain cardiovascular drugs (25% vs. 23 and 22%), and anxiolytics/antidepressants (47% vs. 43 and 43%) (all *p* < 0.05; Fig. 3).

### Healthcare provider type

Claims for laboratory tests do not indicate the type of provider who ordered the test, so provider types are only reported for the strictly-defined and the broadly-defined HCU cohorts and not reported for the elevated tHcy cohort (Table 2). Among patients with > 2 HCU diagnoses, including 1 ICD-10 diagnosis (strictly-defined HCU) or > 1 ICD-10 HCU diagnosis (broadly- defined HCU) 37 and 38%, respectively, received the index diagnosis claim from a family medicine physician, and 21% of patients in both groups received that diagnosis from an internal medicine physician. Very few (strict HCU: 0.2%; broad HCU: 0.1%) patients with an HCU diagnosis had a genetics specialist noted on their index claim, and only 62 patients out of 6613 (~ 1%) with an HCU diagnosis had a genetics specialist noted on any healthcare claim during their enrollment in MarketScan between January 1, 2010 and July 31, 2017.

**Table 2:** Provider types on index claims of patients with strictly- and broadly-defined homocystinuria (HCU)

	Strictly-defined HCU^**a**^	Broadly-defined HCU^**b**^
	*N* = 2450	N = 6613
**Provider Types**		
Cardiology	40 (1.6%)	129 (2.0%)
Endocrinology	25 (1.0%)	51 (0.8%)
Family medicine	898 (36.7%)	2483 (37.5%)
Genetics	6 (0.2%)	9 (0.1%)
Healthcare facility	262 (10.7%)	747 (11.3%)
Hematology	79 (3.2%)	162 (2.4%)
Internal medicine	525 (21.4%)	1378 (20.8%)
Neurology	164 (6.7%)	327 (4.9%)
Nurse or PA	79 (3.2%)	256 (3.9%)
OB/GYN	42 (1.7%)	148 (2.2%)
Oncology	68 (2.8%)	159 (2.4%)
Pediatrics	19 (0.8%)	46 (0.7%)
Psychiatry	10 (0.4%)	27 (0.4%)
Surgery	28 (1.1%)	54 (0.8%)
Unspecified MD	112 (4.6%)	312 (4.7%)
Other^c^	101 (4.1%)	344 (5.2%)

^a^ > 2 HCU diagnoses, including > 1 ICD-10 diagnosis; ^b^ > 1 ICD-10 HCU diagnosis; ^c^ Patients with > 1 claim with an HCU diagnosis code on their index date may be included in > 1 provider type category; *HCU* homocystinuria, *MD* Doctor of Medicine, *OB/GYN* obstetrician/gynecologist, *PA* physician’s assistant

### Healthcare resource utilization and costs

Patients with broadly-defined HCU and those with elevated tHcy had 3–4 times as many IP admissions PPPM (broadly-defined HCU: 0.04 [0.49]; elevated tHcy: 0.03 [0.17]), compared to PKU patients (0.01 [0.08]; *p* < 0.05), and more than twice as many days of hospitalization PPPM (0.17 [1.70], 0.22 [1.35], and 0.08 [0.68], respectively; *p* < 0.05). The frequency of ER visits was up to 44% higher among broadly-defined HCU and elevated tHcy patients (0.05 [0.29] and 0.06 [0.17] visits PPPM), compared to PKU patients (0.04 [0.27]; *p* < 0.05). Patients with diagnosed HCU and patients with elevated tHcy had up to 50% more OP office visits PPPM (strictly-defined HCU: 0.81 [0.69]; broadly-defined HCU: 0.97 [1.23]; elevated tHcy: 0.89 [1.84]), compared to PKU patients (0.65 [1.56]; p < 0.05). Similar patterns were observed for lab tests, other OP visits, and OP prescriptions (Table 3).

**Table 3:** Healthcare use and costs among patients with diagnosed HCU, elevated tHcy without HCU, and PKU

	Strictly-defined HCU^**a**^		Broadly-defined HCU^**b**^		Elevated tHcy^**c**^		PKU^**d**^	
	N = 2450		***N*** = 6613		N = 2017		N = 5120	
	N (%) or Mean (SD)	Median	N (%) or Mean (SD)	Median	N (%) or Mean (SD)	Median	N (%) or Mean (SD)	Median
**Months of follow-up**	23.5 (16.4)*^	19.0	12.7 (13.4)*^	9.2	14.5 (14.5)*	9.4	26.4 (22.1)	19.9
**Inpatient (IP) admissions**								
Patients with > 1 IP admission	444 (18.1%)*	–	892 (13.5%)^	–	393 (19.5%)*	–	630 (12.3%)	–
Number of IP admissions PPPM^e^	0.01 (0.06)^	0.0	0.04 (0.49)*	0.0	0.03 (0.17)*	0.0	0.01 (0.08)	0.0
Total days of hospitalization PPPM	0.07 (0.42)^	0.0	0.17 (1.70)*	0.0	0.22 (1.35)*	0.0	0.08 (0.68)	0.0
IP costs PPPM	$385 ($2937)^	$0	$1191 ($26,270)*	$0	$605 ($2534)	$0	$407 ($4564)	$0
**Emergency room (ER) visits**								
Patients with > 1 ER visit	754 (30.8%)	–	1349 (20.4%)*^	–	570 (28.3%)*	–	1576 (30.8%)	–
Number of ER visits PPPM	0.04 (0.13)^	0.0	0.05 (0.29)*	0.0	0.06 (0.17)*	0.0	0.04 (0.27)	0.0
ER costs PPPM	$66 ($277)*	$0	$85 ($514)*	$0	$80 ($350)*	$0	$39 ($178)	$0
**Outpatient (OP) office visits**								
Patients with > 1 OP office visit	2422 (98.9%)*^	–	6354 (96.1%)*^	–	1840 (91.2%)*	–	4865 (95.0%)	–
Number of OP office visits PPPM	0.81 (0.69)*^	0.6	0.97 (1.23)*^	0.7	0.89 (1.84)*	0.6	0.65 (1.56)	0.4
OP office visit costs PPPM	$90 ($103)	$66	$113 ($175)*^	$70	$87 ($109)	$60	$89 ($202)	$52
**Lab tests**								
Patients with > 1 OP lab test	2301 (93.9%)*	–	5329 (80.6%)*^	–	1905 (94.4%)*	–	4469 (87.3%)	–
Number of OP lab tests PPPM	2.2 (3.2)*^	1.4	2.4 (5.5)*^	1.2	5.3 (19.6)*	2.5	1.2 (3.6)	0.4
OP lab test costs PPPM	$73 ($198)*	$28	$84 ($333)*	$20	$92 ($457)*	$32	$47 ($176)	$8
**Other OP visits**								
Patients with > 1 other OP visit	2413 (98.5%)*^	–	6061 (91.7%)*^	–	1927 (95.5%)	–	4868 (95.1%)	–
Number of other OP visits PPPM	1.4 (1.9)*^	0.9	1.5 (2.3)*^	0.8	1.8 (3.6)*	0.9	1.0 (4.8)	0.5
Other OP costs PPPM	$647 ($2464)*	$190	$751 ($3079)*	$155	$709 ($1984)^+^	$150	$477 ($3838)	$81
**OP prescriptions**								
Patients with > 1 OP prescription	2345 (95.7%)*^	–	5893 (89.1%)*^	–	1850 (91.7%)*		4147 (81.0%)	–
Number of OP prescriptions PPPM	2.5 (2.2)*^	1.9	2.5 (3.1)*^	1.8	2.8 (2.8)*	2.1	1.0 (2.4)	0.4
OP prescription costs PPPM	$366 ($1016)*	$107	$347 ($1214)*	$76	$313 ($746)*	$80	$1063 ($4186)	$12
**Total medical costs**^**f**^**PPPM**	$1262 ($4353)^	$385	$2224 ($26,689)*	$358	$1573 ($3804)*	$369	$1060 ($6777)	$243
**Total healthcare costs**^**g**^**PPPM**	$1629 ($4753)*	$614	$2570 ($26,758)	$576	$1886 ($3969)	$579	$2123 ($7987)	$365

^a^ > 2 HCU diagnoses, including > 1 ICD-10 diagnosis; ^b^ > 1 ICD-10 HCU diagnosis; ^c^ tHcy lab result > 20 μmol/L; ^d^ > 1 ICD-9 or ICD-10 PKU diagnosis; ^e^ Mean (SD) and median number of services and costs calculated among all patients, including those who did and did not have > 1 of that healthcare service during follow-up; ^f^ All IP and OP medical services (not including outpatient prescriptions); ^g^ Medical + outpatient prescriptions; *significantly different from PKU, *p* < 0.05; ^significantly different from elevated tHcy, *p* < 0.05; *SD* standard deviation, *PPPM* per patient per month

**Fig. 4:**
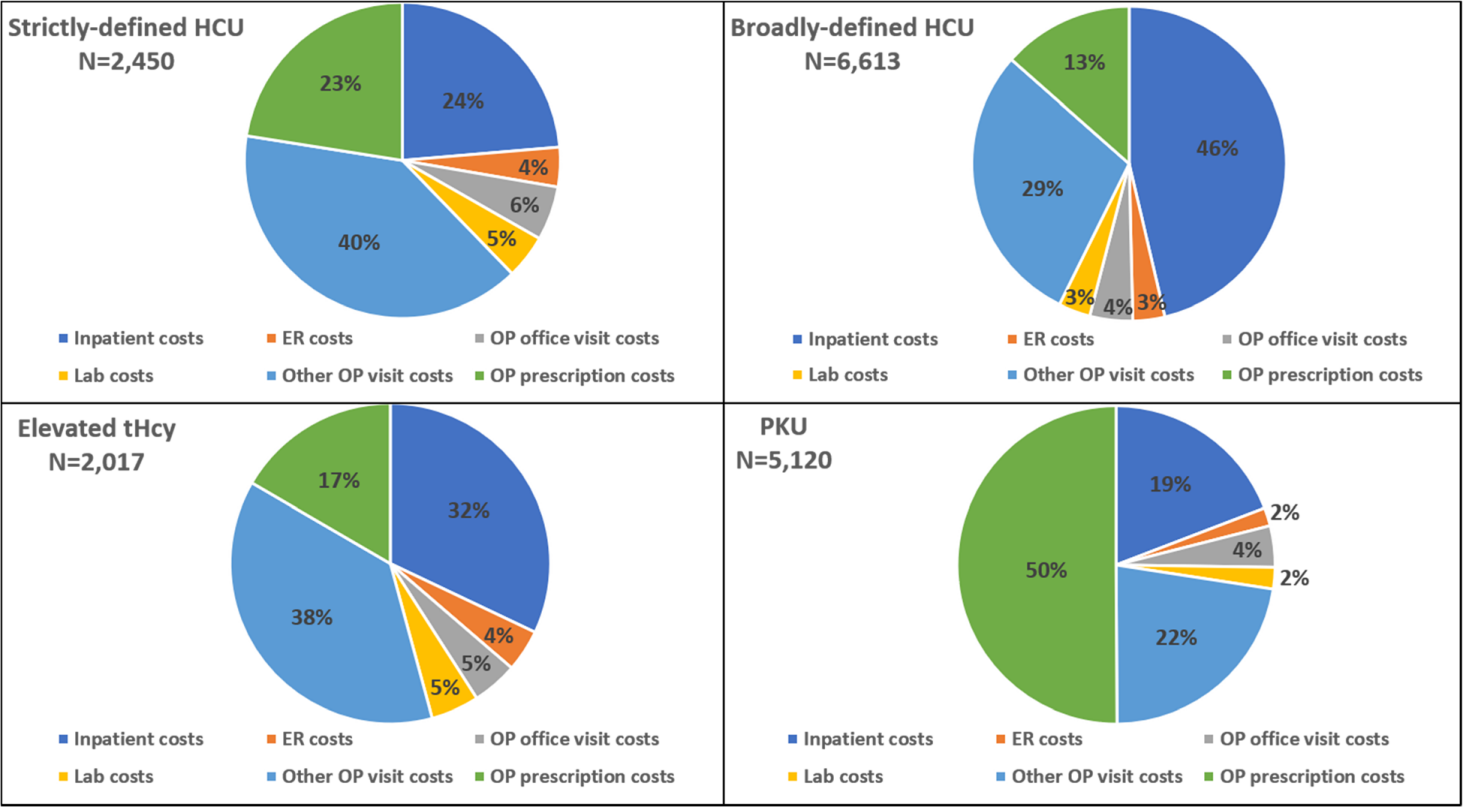
Proportion of costs attributable to different healthcare services. ER: emergency room; HCU: homocystinuria; OP: outpatient; PKU: phenylketonuria; tHcy: total homocysteine

Figure 4 depicts the difference between cohorts in the proportion of total healthcare costs contributed by each healthcare service type (IP, ER, OP office visits, lab, other OP visits, prescriptions). The proportion of total healthcare costs due to IP admissions was significantly higher among patients with strictly-defined HCU (24%), broadly-defined HCU (46%), and elevated tHcy (32%), compared to PKU patients (19%; all *p* < 0.05). Other OP visits contributed the highest proportion of total costs for patients with strictly-defined HCU (40%) and elevated tHcy (38%). Half of all healthcare costs among PKU patients were for OP prescriptions.

Observed healthcare costs were higher among broadly-defined HCU patients, compared to PKU patients, for all service categories except OP prescriptions (Table 3). Total healthcare costs PPPM were highest among broadly-defined HCU patients ($2570 [$26,758]), though not significantly different from PKU ($2123 [$7987]; *p* = 0.25).

Strictly-defined HCU costs were similar or higher than PKU for all costs except for OP prescription costs. Thus, total healthcare costs PPPM among patients with strictly-defined HCU were significantly lower than PKU patients ($1629 [$4753] vs. $2123 [$7987]; *p* < 0.05), but similar to patients with elevated tHcy ($1886 [$3969]; *p* = 0.05). This difference was largely driven by lower OP prescriptions costs among strictly-defined HCU and elevated tHcy patients, compared to PKU patients. Notably, total non-prescription costs (i.e., medical costs) among patients with broadly-defined HCU were more than twice that of PKU patients ($2224 [$26,689] vs. $1060 [$6777]; *p* < 0.05), and non-prescription costs were nearly 50% higher among patients with elevated tHcy ($1573 [$3804), compared to PKU (p < 0.05)

### National Prevalence Estimates

To estimate the national prevalence of diagnosed HCU, elevated tHcy, and diagnosed PKU, rates per 1000 of strictly- and broadly-defined HCU, elevated tHcy without an HCU diagnosis, and diagnosed PKU in MarketScan were applied to the overall US population, and to US population age groups.

The projected prevalence of diagnosed HCU ranged from 12,113 (95% CI: 11,634-12,593) with the strict definition to 31,162 (30,411-31,913) with the broad definition of HCU. Age-adjusted prevalence of elevated tHcy without an HCU diagnosis was estimated to be 89,470 (85,565-93,374). Approximately 16,615 (16,160-17,070) patients were projected to be diagnosed with PKU in the US (Table 4).

**Table 4:** Projected US prevalence of diagnosed HCU, elevated tHcy without HCU, and diagnosed PKU

	US Population^a^			
	N = 323,127,513			
	Strictly-defined HCU^b^	Broadly-defined HCU^c^	Elevated tHcy^d^	PKU^e^
**Unadjusted prevalence**^**f**^**(N, 95% CI)**	8137 (7815–8459)	21,963 (21434–22,493)	85,758 (82015–89,500)	17,005 (16539–17,471)
**Age-adjusted prevalence (N, 95% CI)**	12,113 (11634–12,593)	31,162 (30411–31,913)	89,470 (85565–93,374)	16,615 (16160–17,070)

^a^US Census Bureau estimate of the US resident population on July 1, 2016; ^b^ > 2 HCU diagnoses, including > 1 ICD-10 diagnosis; ^c^ > 1 ICD-10 HCU diagnosis; ^d^ tHcy lab result > 20 μmol/L; ^e^ > 1 ICD-9 or ICD-10 PKU diagnosis; ^f^ Prevalence estimates are for 1/1/2017 and assume that the incidence and duration of HCU and PKU remained constant throughout the study period (1/1/2010–12/31/2016); CI: confidence interval

## Discussion

Despite both HCU and PKU being genetic inborn errors of amino acid metabolism, the age of the diagnosed HCU patients in this retrospective analysis of healthcare claims suggests that patients with HCU are diagnosed much later in life. As expected, the highest proportion of diagnosed PKU patients was observed in the youngest age group (0–11 years), likely due to infants being diagnosed through effective universal newborn screening. Conversely, the prevalence of diagnosed HCU among younger patients was dramatically lower than among older individuals, implying that HCU patients are not diagnosed primarily at birth or during early childhood, even though HCU is a lifelong genetic disease. The greater frequency of homocysteine testing in middle aged and older patients than in younger patients contributes to this diagnostic pattern. These data suggest that newborn screening fails to capture the vast majority of HCU cases, often leaving patients without a diagnosis until late adulthood when they present with serious symptoms or comorbid conditions indicative of HCU.

Several conditions other than HCU can lead to elevated tHcy levels, such as kidney disease/failure, megaloblastic anemia, and hypothyroidism. All of these were meaningfully and statistically more common among patients with elevated tHcy and no HCU diagnosis compared to patients with strictly- or broadly defined HCU, indicating the specificity of the HCU ICD-10 diagnostic code.

Patients whose HCU remains undetected for decades may develop comorbidities associated with elevated tHcy, such as those observed with high frequency in this study, including hyperlipidemia, hypertension, painful joints and limbs, and vitamin deficiencies, among others. In addition to the high prevalence of various comorbid conditions, patients with an HCU diagnosis or elevated tHcy had higher rates of all-cause healthcare utilization in all service categories captured in this study, compared to their PKU counterparts. Specifically, diagnosed HCU patients and those with elevated tHcy without an HCU diagnosis had 3–4 times the rate of hospitalizations and more than twice as many total days of hospitalization PPPM than patients with PKU. In turn, IP costs contributed significantly more to overall healthcare costs among patients with diagnosed HCU and elevated tHcy, compared to PKU patients. It should be noted that a two-part multivariable model found no significant difference in IP costs (representing the largest unadjusted difference between groups) between broadly-defined HCU and PKU patients, after controlling for patient characteristics (age, sex, urban residence, geographic region, insurance type, length of follow-up). It is also worth noting, however, that the actual healthcare costs paid (unadjusted) for IP and OP medical services among patients with a broadly-defined HCU diagnosis or elevated tHcy were 48 to > 100% higher than the medical costs of patients with PKU.

The projected national prevalence of 16,615 patients with PKU from this analysis is in line with the latest estimate of 16,500 reported by the National PKU Alliance [11]. Based on the projections from this study, however, the actual prevalence of HCU in the US may be 10 or more times current estimates of HCU prevalence [4–6], and two or more times the prevalence of the more easily detected and widely recognized condition of PKU. These projections point to a non-trivial number of patients living with undiagnosed HCU early in life and suffering from various sequela of the disease without awareness of what is causing their symptoms. Given that the majority of HCU claims observed in this study were submitted by family and internal medicine practitioners, improving primary care providers’ understanding and management of HCU may lead to earlier diagnosis and lessen the healthcare utilization and costs associated with the condition, including resource-intensive and costly hospitalizations. Better HCU screening and more timely diagnosis would allow for adherence to treatment guidelines [12] across the spectrum of healthcare providers and could contribute to reduced burden of the disease on patients and reduced economic burden on the healthcare system.

### Limitations

This study was subject to some limitations that are inherent in claims-based analyses. First, this study was limited to only those individuals with commercial health insurance coverage or private Medicare supplemental coverage, and so the results of this analysis may not be generalizable to individuals with other insurance or without health insurance coverage. Additionally, it should be noted that the index date for each patient was identified as the first claim with HCU or PKU diagnosis, or first elevated tHcy lab test result, observed between January 1, 2010 and December 31, 2016 (all available data); these dates do not necessarily represent the first time patients were diagnosed with HCU or PKU, or their first elevated tHcy test. This means the study sample is a mix of incident and prevalent cases. The data in this study, which are limited to that found in administrative claims, may also be subject to data coding limitations and data entry error which may lead to misclassification of diagnoses included in the study.

Finally, most of the healthcare cost data presented here, and the comparisons made between groups, do not account for differences between groups in patient characteristics such as age and comorbidities, which may impact healthcare utilization and expenditures. When differences in IP costs between broadly-defined HCU and PKU patients were examined in a multivariate model the differences seen were no longer statistically significant. The unadjusted costs do, however, represent the actual amounts paid for healthcare services among patients with diagnosed HCU, elevated tHcy, and diagnosed PKU.

## Conclusion

The results of this study show that the actual prevalence of HCU may be 10 or more times current estimates of HCU prevalence, at 1 in 10,000 individuals in the US. Newborn screening for HCU fails to capture the vast majority of HCU cases, and many individuals in the US may be underdiagnosed or misdiagnosed until late in life, if at all. Ideally, patients with HCU should be diagnosed as early as possible to minimize the symptoms and comorbidities associated with HCU, and so improvements to newborn screening and detection in the pediatric population may be warranted. Enhanced education of physicians regarding the diagnosis and management of HCU may also be necessary. Taking steps toward improved detection and treatment of HCU could help reduce the burden of the disease among the 12,000 to 30,000 Americans that may be affected.

## Data Availability

The data that support the findings of this study are available from IBM Watson Health, but restrictions apply. These data were used under license for the current study, and so are not publicly available. Data are, however, available from the authors upon reasonable request and with permission of IBM Watson Health.

## Abbreviations

CBS: Cystathionine beta synthase
CI: Confidence interval
EPO: Exclusive provider organization
ER: Emergency room
HCU: Homocystinuria
ICD: International classification of diseases
IP: Inpatient
MD: Doctor of Medicine
OB/GYN: Obstetrician/gynecologist
OP: Outpatient
PA: Physician’s assistant
PKU: Phenylketonuria
POS: Point of service
PPO: Preferred provider organization
PPPM: Per patient per month
SD: Standard deviation
tHcy: Total homocysteine
US: United States
